# Speech Emotion Recognition Using Convolution Neural Networks and Multi-Head Convolutional Transformer

**DOI:** 10.3390/s23136212

**Published:** 2023-07-07

**Authors:** Rizwan Ullah, Muhammad Asif, Wahab Ali Shah, Fakhar Anjam, Ibrar Ullah, Tahir Khurshaid, Lunchakorn Wuttisittikulkij, Shashi Shah, Syed Mansoor Ali, Mohammad Alibakhshikenari

**Affiliations:** 1 Wireless Communication Ecosystem Research Unit, Department of Electrical Engineering, Chulalongkorn University, Bangkok 10330, Thailandshah.shashi415@gmail.com (S.S.); 2 Department of Electrical Engineering, Main Campus, University of Science & Technology, Bannu 28100, Pakistan; masifeed@ustb.edu.pk (M.A.); F.Anjam@ustb.edu.pk (F.A.); 3 Department of Electrical Engineering, Namal University, Mianwali 42250, Pakistan; 4 Department of Electrical Engineering, Kohat Campus, University of Engineering and Technology Peshawar, Kohat 25000, Pakistan; ibrarullah@uetpeshawar.edu.pk; 5 Department of Electrical Engineering, Yeungnam University, Gyeongsan 38541, Republic of Korea; 6 Department of Physics and Astronomy, College of Science, King Saud University, P.O. Box 2455, Riyadh 11451, Saudi Arabia; symali@ksu.edu.sa; 7 Department of Signal Theory and Communications, Universidad Carlos III de Madrid, Leganés, 28911 Madrid, Spain

**Keywords:** speech emotion recognition, convolutional neural networks, convolutional Transformer encoder, multi-head attention, spatial features, temporal features

## Abstract

Speech emotion recognition (SER) is a challenging task in human–computer interaction (HCI) systems. One of the key challenges in speech emotion recognition is to extract the emotional features effectively from a speech utterance. Despite the promising results of recent studies, they generally do not leverage advanced fusion algorithms for the generation of effective representations of emotional features in speech utterances. To address this problem, we describe the fusion of spatial and temporal feature representations of speech emotion by parallelizing convolutional neural networks (CNNs) and a Transformer encoder for SER. We stack two parallel CNNs for spatial feature representation in parallel to a Transformer encoder for temporal feature representation, thereby simultaneously expanding the filter depth and reducing the feature map with an expressive hierarchical feature representation at a lower computational cost. We use the RAVDESS dataset to recognize eight different speech emotions. We augment and intensify the variations in the dataset to minimize model overfitting. Additive White Gaussian Noise (AWGN) is used to augment the RAVDESS dataset. With the spatial and sequential feature representations of CNNs and the Transformer, the SER model achieves 82.31% accuracy for eight emotions on a hold-out dataset. In addition, the SER system is evaluated with the IEMOCAP dataset and achieves 79.42% recognition accuracy for five emotions. Experimental results on the RAVDESS and IEMOCAP datasets show the success of the presented SER system and demonstrate an absolute performance improvement over the state-of-the-art (SOTA) models.

## 1. Introduction

In the context of rapidly advancing Artificial Intelligence (AI), human–computer interactions (HCI) are studied in depth. We are living in a world where Siri and Alexa are physically closer. Understanding human emotions paves the way toward understanding people’s needs. Speech emotion recognition (SER) systems [1] classify emotions in speech utterances and are vital in advancing the HCI, healthcare, customer satisfaction, social media analysis, stress monitoring, and intelligent systems. Moreover, SER systems are useful in online tutorials, language translation, intelligent driving, and therapy sessions. In a few situations, humans can be substituted by computer-generated characters with the ability to act naturally and communicate convincingly by expressing human-like emotions. Machines need to interpret the emotions carried by speech utterances. Only with such an ability can a completely expressive dialogue based on joint human–machine trust and understanding be accomplished.

## 2. Problems and Motivations

SER is a challenging task due to various reasons. Firstly, (i) emotions are subjective and their expression can vary significantly across individuals. Different people may exhibit varying patterns of speech, tone, and vocal cues to convey the same emotion. (ii) The availability of high-quality, diverse, and standardized datasets is crucial in training and evaluating SER models. (iii) Emotions are often context-dependent, and the same speech utterance can convey different emotions depending on the situational context. These problems are considered the motivations of this study to obtain better SER results. To address the first problem, the proposed model uses MFCCs as input regardless of the patterns of speech and tones. The MFCCs’ spectrograms can serve as a solution to this problem. Data augmentation can be utilized to address the data scarcity problem. We augment the dataset by adding white noise to the speech signals such that the database size is increased. To address the third problem, the model uses a Transformer encoder module to obtain the context-dependent (temporal) features.

Speech emotion recognition systems have gained attention due to the extensive use of deep learning. Prior to deep learning, SER systems were reliant on techniques such as hidden Markov models (HMM) [2], Gaussian mixture models (GMM) [3], and support vector machines (SVM) [4], along with extensive preprocessing and accurate feature engineering. Comprehensive reviews of SER systems are available in [5,6]. A benchmark comparison is available in [7]. However, the development of deep learning tools and processes, and solutions for SER, has also changed. There have been significant studies and research proposing SER techniques to recognize and classify various emotions in speech [8,9,10,11,12,13,14]. In addition to recent developments in deep learning, there has been a wave of studies on SER using long short-term memory, recurrent neural networks, generative adversarial networks, and autoencoders to solve the above problem [15,16,17,18,19,20,21,22].

In the recent past, deep learning has significantly contributed to natural language understanding (NLU). Deep belief network (DBN)-based SER in [23,24] showed a substantial improvement over the baseline non-DL models [25]. Extreme learning machine (ELM)-based SER in [26,27] used feature representations from the probability distributions at the segment level, employing a single hidden layer neural network to classify speech emotions at the utterance level. Deep hierarchical models, data augmentation, and regularization-based DNNs for SER are proposed in [28], whereas deep CNNs using spectrograms are proposed in [29]. DNNs are trained for SER with the acoustic features extracted from the short intervals of speech using a probabilistic CTC loss function in [30]. Bidirectional LSTM-based SER in [31] is trained on feature sequences and achieves better accuracy than DNN-ELM [26]. Deep CNN+LSTM-based SER in [32] achieves even better results. The hybrid deep CNN + LSTM improves the SER accuracy but raises the overall computational complexity. Auditory–visual modality (AVM)-based SER in [33] captures emotional content from different speaking styles. The Tensor Fusion Network (TFN)-based SER in [34] learns intra- and inter-modality dynamics. Convolutional deep belief network-based SER in [35] learns multimodal feature representations linked to expressions. The single plain CNN model is weak in classifying the speaker’s emotional state with the required accuracy level because it loses some basic sequential information during the convolutional operation. Therefore, two parallel CNN models can solve the limitation concerning the loss of important information in speech. The study in [36] shows two parallel CNN models and utilizes them for SER accordingly.

With dominance, pleasure, and excitement, one can nearly define all emotions; however, the implementation of such a deterministic system using DL is very challenging and complex. Therefore, in DL, statistical models and the clustering of samples are used to qualitatively classify emotions such as sadness, happiness, and anger. For the classification and clustering of emotions, features must be extracted from speech, usually relying on different types of prosody, voice quality, and spectral features [37]. The prosody features usually include the fundamental frequency (F0), intensity, and speaking rate, but they cannot confidently discriminate between angry and happy emotions. The features associated with voice quality are usually the most successful in determining the emotions of the same speaker. However, these features vary from speaker to speaker, making them difficult to use in speaker-independent settings [38]. On the other hand, spectral features are widely used to determine emotions from speech. These features can confidently distinguish anger from happiness. However, the magnitudes and shifts of the formant frequencies for identical emotions change across different vowels, which increases the complexity of the speech emotion recognition system [39]. For all the feature types, there are several standard representations of features. Prosody features are typically represented by F0 and measure the speaking rates [40], whereas spectral features are defined by cepstrum-based feature representations. Mel-frequency cepstral coefficients (MFCC) or linear prediction cepstral coefficients (LPCC) are commonly used spectral features along with formants, and other information can also be used [41]. Finally, the voice quality features usually include the normalized amplitude quotient, shimmer, and jitter [42].

Feature extraction is a crucial step in many machine learning tasks, including speech recognition, computer vision, and natural language processing. The goal of feature extraction is to transform raw data into a representation that captures the most salient information for the task at hand. In speech recognition, features are typically extracted from the acoustic signal using techniques such as mel-frequency cepstral coefficients (MFCCs), which have been widely used in the literature due to their effectiveness in capturing the spectral envelope of a signal. Other popular techniques include perceptual linear predictive (PLP) features, gamma tone features, and filterbank energies. In computer vision, features are extracted from images using techniques such as SIFT, SURF, and HOG, which are effective in capturing local visual patterns. In natural language processing, features are extracted from text using techniques such as bag-of-words, n-grams, and word embeddings, which capture the syntactic and semantic information in the text [43,44,45,46,47,48]. This study uses MFCCs as input features for several reasons. First, (i) the MFCCs are used as a grayscale image as a simultaneous input to the parallel CNNs and Transformer modules for spectral and temporal feature extraction. (ii) MFCCs can capture the spectral envelopes of speech signals, which is crucial in characterizing different emotional states. MFCCs are less sensitive to variations in speaker characteristics, background noise, and channel distortions, making them more robust for emotion recognition tasks. (iii) MFCCs are derived based on the human auditory system’s frequency resolution, which aligns well with how humans perceive and differentiate sounds. By focusing on perceptually relevant information, MFCCs can effectively capture the distinctive features related to emotions conveyed through speech. (iv) MFCCs provide a compact representation of speech signals by summarizing the spectral information into a smaller number of coefficients. This dimensionality reduction helps to reduce the computational complexity and memory requirements of SER models while still preserving the essential information needed for emotion classification. (v) By computing MFCCs over short time frames and applying temporal analysis techniques such as delta and delta–delta features, the dynamic changes in speech can be captured. Emotions often manifest as temporal patterns in speech, and MFCCs enable the modeling of these dynamics, enhancing the discriminative power of SER models.

We have studied and examined the recent speech processing literature and observed that speech signals follow a hybrid structure, such as temporal features and spatial features, where both feature representations contain essential cues for SER. The majority of the existing SER systems lack parallel neural architectures to process speech signals and acquire information about high-level deep spatiotemporal features. As a result of this limitation, we have proposed a fusion of spatial and temporal feature representations of speech emotions by parallelizing CNNs and a Transformer encoder for SER, named CTENet. We have stacked two parallel CNNs for the spatial feature representation, which is paired with multi-head self-attention layers from the Transformer encoder for the temporal feature representation to classify the speech emotions. By increasing the filter channel dimensions and decreasing the feature maps of CNNs, better feature representations can be achieved at a low computational cost. The Transformer encoder is utilized such that the SER model can learn to anticipate the frequency distributions of various speech emotions. The MFCC plot of a speech utterance is treated as a grayscale image where the width of the MFCC is treated as a time scale and the height is treated as a frequency scale, respectively. The pixel values in the MFCC plots are the speech signal intensities at the mel-frequency range and time steps. Since the input data are sequential, the Transformer accurately establishes the temporal relations between the pitch transitions in various emotions. We augment and intensify the variations in the RAVDESS dataset with AWGN to minimize model overfitting. With the CNN for the spatial feature representations and the Transformer for the sequential encoding, the proposed CTENet for SER achieves 82.31% accuracy when classifying eight speech emotions. The main contributions of this study are summarized below.

- Stacked parallel CNNs with multi-head self-attention layers are implemented. The channel dimensions of filters and feature maps are reduced, allowing an expressive representation of features at a lower computational cost. With multi-head self-attention, the network learns to predict the frequency distributions of speech emotions in accordance with the overall MFCC structure.
- With the classification and spatial feature representation of CNNs, the MFCCs are used as grayscale images, where the widths and heights of the MFCC are the time and frequency scales, respectively. The pixel values in the MFCC indicate the speech signal intensities at the mel-frequency range and time steps.
- The dataset is augmented with AWGN. Creating new, real samples is a very difficult task. Thus, white noise is added to the speech signals to mask the random effect of noise existing in the training dataset. Moreover, this generates pseudo-new training samples and counterbalances the noise impact inherent in the dataset.

The rest of this paper is organized as follows. The related SER literature is presented in Section 2. An in-depth explanation of the proposed SER with parallel CNNs using skips and a Transformer encoder is given in Section 3. The experiments and setups are explained in Section 4. Section 5 gives the results and discussion. Finally, Section 6 concludes this research.

## 3. Related SER Literature

Speech emotion recognition is an attractive research field and numerous novel techniques have been proposed to learn optimal SER solutions. The SER method contains two modules, namely feature representation and emotion classification. Optimal feature representation and superior classification for a robust SER system are difficult tasks [9]. The MFCC feature-based SER in [49] classifies various emotions using the logistic model tree (LMT) classifier. An ensemble model using 20 SVMs with a Gaussian kernel in [50] is proposed for SER and achieves 75.79% accuracy. The 2D-CNN-based SER method in [51] recognizes emotions by extracting deep discriminative cues from spectrograms. Pre-trained CNN architectures—for example, AlexNet and VGG—are used to construct the SER framework via transfer learning to classify emotions from spectrograms in [52]. A trained CNN model in [53] is utilized for the extraction of features from spectrograms, and speech emotions are classified using SVM. Moreover, 1D-CNN + FCN-based SER in [54] use prosodic and spectral features from MFCCs to classify various speech emotions. The LSTM and RNNs are used to classify the long-term sequences in the speech signals for SER [55]. The DNN-LSTM-based SER method in [56] uses a hybrid approach to learn spatiotemporal cues from raw speech data.

The CNN-BLSTM-based SER method in [57] learns the spatial features and temporal cues of speech symbols and increases the accuracy of the existing model. The SER extracts spatial features and feeds them to the BLSTM in order to learn temporal cues for the recognition of the emotional state. A DNN in [26] is used to compute the probability distributions for various emotions given all segments. The DNN identifies emotions from utterance-level feature representations, and, with the given features, ELM is used to classify speech emotions. The CNN in [58] successfully detects emotions with 66.1% accuracy when compared to the feature-based SVM. Meanwhile, the 1D-CNN in [59] reports 96.60% classification accuracy for negative emotions. The CNN-based SER in [60] learns deep features and employs a plain rectangular filter with a new pooling scheme to achieve more effective emotion discrimination. A novel attention-based SER is proposed utilizing a long attention process to link mel-spectrogram and interspeech-09 features to generate the attention weights for a CNN. A deep CNN-based SER is constructed in [61] for the ImageNet LSVRC-2010 challenge. The AlexNet trained with 1.2 million images and fine-tuned with samples from the EMO-DB is used to recognize angry, sad, and happy emotions. An end-to-end context-aware SER system in [62] classifies speech emotions using CNNs followed by LSTM.

The difference compared to other deep learning SER frameworks lies in not using the preselected features before network training and introducing raw input to the SER system. The ConvLSTM-based SER in [63] adopted convolutional LSTM layers for the state transitions so as to extract spatial cues. Four LFLBs are used for the extraction of the spatiotemporal cues in the hierarchical correlational form of speech signals utilizing a residual learning strategy. The BLSTM + CNN stacking-based SER in [64] matches the input formats and recognizes emotions by using logistic regression. BC-LSTM relies on context-aware utterance-level representations of features. This model captures the contextual cues from utterances using a BLSTM layer. The SVM-DBN-based SER in [65] improves emotion recognition via diverse feature representation. Gender-dependent and -independent results show 80.11% accuracy. The deep-stride CNN-based SER in [66] uses raw spectrograms and learns discriminative features from speech spectrograms. After learning the features, the Softmax classifier is employed to classify speech emotions.

Attention mechanism-based deep learning for SER is another notable approach that has achieved vast success; a complete review can be found in [67]. In classical DL-based SER, all features in a given utterance receive the same attention. Nevertheless, emotions are not consistently distributed over all localities in the speech samples. In attention-based DL, attention is paid by the classifier to the given specific localities of the samples using attention weights assigned to a particular locality of data. The SER system based on multi-layer perceptron (MLP) and a dilated CNN in [68] uses channel and spatial attention to extract cues from input tensors. Bidirectional LSTM with the weighted-polling scheme in [69] learns more illustrative feature representations concerning speech emotions. The model focuses more on the main emotional aspects of an utterance, whereas it ignores other aspects of the utterance. The self-attention and multitasking learning CNN-BLSTM in [70] improves the SER accuracy by 7.7% in comparison with the multi-channel CNN [71] when applied to the IEMOCAP dataset. With speech spectrograms as input, gender classification has been considered as a secondary task. The LSTM in [18] for SER demonstrates reduced computational complexity by replacing the LSTM forget gate with an attention gate, where attention is applied on the time and feature dimensions. The attention LSTM-based time-delay SER in [72] extracts high-level feature representations from raw speech waveforms to classify emotions.

The deep RNN-based SER in [73] learns emotionally related acoustic features and aggregates them temporally into a compact representation at the utterance level. Another deep CNN [74] is proposed for SER. In addition, a feature pooling strategy over time is proposed, using local attention to focus on specific localities of a speech utterance that are emotionally prominent. A self-attention mechanism utilizes a CNN via sequential learning to generate the attention weights. Another attention-based SER is proposed that uses a fully connected neural network (FCNN). Frame- and utterance-level features are used for emotion classification by applying MLP and attention processes to classify emotions. A multi-hop attention model for SER in [75] uses two BLSTM streams to extract the hidden cues from speech utterances. The multi-hop attention model is applied for the generation of final weights for the classification of emotions. Other important research related to SER includes fake news and sentiment analysis, as emotions can also be found in fake news, negative sentiments, and hate speech [76,77,78,79,80,81]. A short summary of the related literature is given in Table 1. Accuracy holds significant importance in the speech emotion recognition (SER) system, where the primary goal is to predict emotions in speech utterances with a high level of precision. Consequently, researchers in the field strive to enhance this particular aspect. By examining Table 1, which is extracted from the aforementioned literature, it becomes evident that models have made advancements in terms of accuracy. However, there is still substantial room for further improvement. Simultaneously, the depth of the model (its computational complexity) remains a crucial consideration for real-time applications. Hence, our objective is to propose an SER model that achieves both high accuracy and a compact size. To accomplish this, we present a novel approach distinct from the models presented in the table, where CNNs combined with RNNs are predominantly employed for SER. Instead, we incorporate Transformer encoders to obtain robust features for network training, as they exhibit strong capabilities in capturing temporal features.

## 4. CTENet SER System

**Figure 1:**
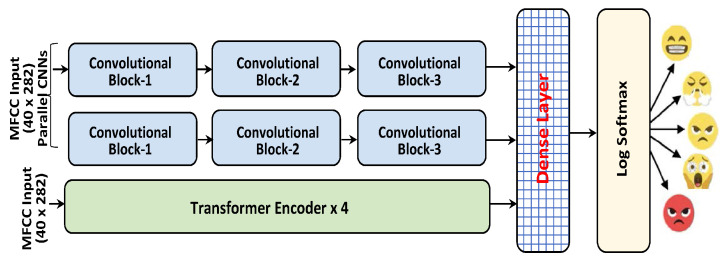
Proposed SER framework: Two parallel CNNs with Transformer encoder for feature extraction. The extracted features are fed to the dense layer with a log Softmax classifier for emotional state prediction.

This section demonstrates the proposed framework and its related modules for speech signals with two parallel CNNs and a multi-head attention Transformer encoder to recognize emotions in speech spectrograms, as described in Figure 1. The suggested SER model comprises three branches, including two CNN modules with skip connections (CNN-Skip), a multi-head attention Transformer encoder module (MTE), and a fully connected dense network (FCDN), to recognize speech signal emotions.

### 4.1. Parallel CNN Framework

**Figure 2:**
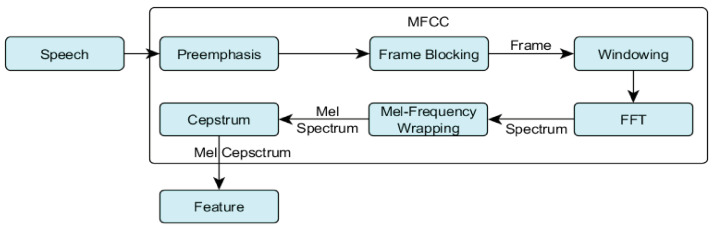
Feature extraction process from input speech signals to MFCC features.

A CNN with 2D-Conv layers is a standard model that accepts input feature maps in terms of batch size, channel, height, and width. The RAVDESS dataset used for training contains 4320 MFCC spectrograms, including 1440 original and 2880 noise-augmented spectrograms. The MFCC feature extraction for model training is depicted in Figure 2. The shape of all MFCCs is (282 × 40), where 40 coefficients represent different ranges of mel pitches with 282 timesteps for every coefficient. The MFCC spectrograms are supposed to be grayscale images with 1 signal intensity channel. The dimensions of the tensor for the MFCC input feature are batch = 4320, channel = 1, height = 40, and width = 282 prior to splitting for training. The activation map is produced after applying the activation function. The kernel size in parallel CNN layers is (3 × 3). The first layer contains a single input channel constructing a filter of (1 × 3 × 3), with 16 output channels imposing 16 filters (1 × 3 × 3) with 9 weights per filter. The subsequent CNN layer contains 16 input and 32 output channels, respectively, imposing 32 filters (16 × 3 × 3) with 144 weights. The second CNN layer applies 32 individually weighted filters (16 × 3 × 3) to input of (16 × 141 × 20), which is the (2 × 2) max-pooled output of the first CNN layer. This creates an output feature map of (32 × 5 × 35) after (4 × 4) max pooling with stride 4. The last CNN layer contains 32 input channels with a (32 × 3 × 3) filter, and 64 output channels imposing 64 filters with 288 weights per filter. The last CNN layer creates the (64 × 1 × 8) output feature map after (4 × 4) max pooling with stride 4. The simultaneously expanded filter depth and feature map reduction provide an expressive hierarchical feature representation at the lowest computational cost. The input channel dimension determines the sizes of all 3D filters in the CNN layers, whereas the output channel dimension determines the number of unique 3D filters in this CNN layer. Each filter is defined by a unique set of weights, and each filter has its own bias term. An activation map of size (O × O × 1) is generated by convolutions performed on an input of size (I × I × C) by a filter of size (F × F), applied to an input containing C channels and (F × F × C) volume, as demonstrated in Figure 2.

**Figure 3:**
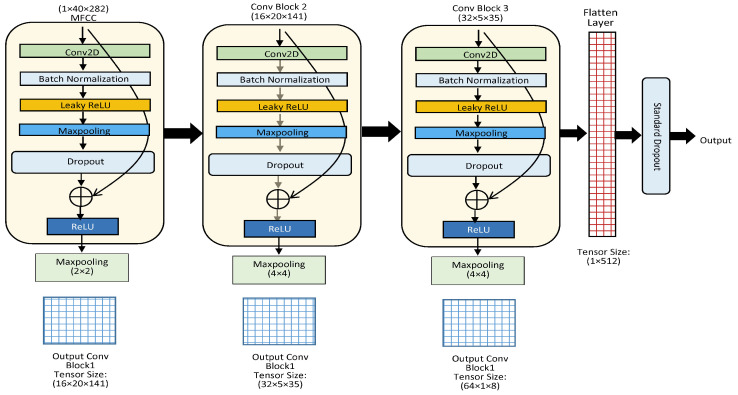
The architecture of a single CNN with skip connections. The proposed model is composed of two parallel CNNs as illustrated in the given architecture.

The gradient becomes very small as the error approaches the prior layers in a very deep architecture. Therefore, to preserve the gradient, skip connections are added to the model as it has been observed that, in prior layers, the learned features correspond to less information extracted from the input. Figure 3 presents the CNN architecture with 3 CNN layers where each block is max-pooled, as well as the skip connection (Figure 3). The parallel CNNs have the same architectural structures as documented above.

### 4.2. Transformer Encoder

The Transformer encoder layer as proposed in [82] is used to anticipate the frequency distributions of various emotions in accordance with the structure of the MFCCs for every emotion. Previously, LSTM-RNNs were used to learn the spectrogram sequences for each emotion and the network only learned to anticipate frequency distributions based on subsequent time steps. Since the emotions cover the complete frequency distributions and not one time step, the multi-head self-attention layers of the Transformer allow the network to seek diversified former time steps while predicting the subsequent ones. The input MFCC features mapped to the Transformer block are max-pooled to considerably decrease the trainable parameters of the network. The context vectors of input sequences are encoded by the Transformer architecture as a set of key (input)–value (input hidden state) pairs (**K**, **V**) with dimensions equivalent to the input sequence length, where keys and values comprise the hidden states of an encoder. The next term in the decoder’s output sequence is a mapping from the **K**–**V** pairs with **Q** as (**Q**, **K**, **V**). The output predicted at the previous time step is computed into a query **Q**. The weighted total of all values from the (**K**, **V**) encoded representation of the inputs reflects the decoder’s outputs. The Transformer’s self-attention gives each hidden state alignment weights as a sequence-length-scaled dot product of the query with all the keys, as follows:

(1) $$Attention\left(Q,K,V\right)=softmax\left(\frac{{\mathrm{QK}}^{T}}{\sqrt{n}}\right)V\;$$

**Figure 4:**
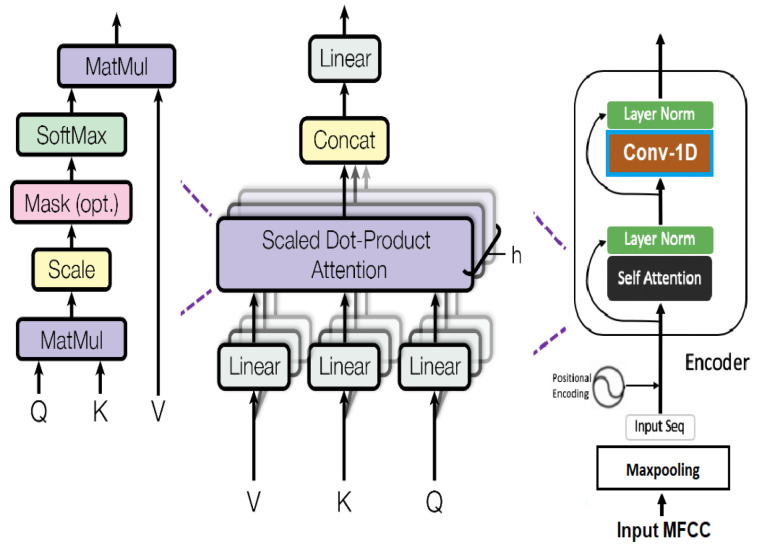
Scaled dot structure with multiple attention heads.

For the sequence output at time step t, the scaled dot product is scaled by dimension n of hidden states. There are various self-attention strategies that can be used. As per [82], the scaled dot product self-attention (**Q**, **K**, **V**) is computed over a number of representation subspaces with a weight matrix specific to each query, key, and value. Multi-head self-attention can compute an output term that is weighted differently based on a subspace of the input sequence in this manner. Concatenating and multiplying the output from each attention head with a weight matrix reduces the dimensions of the encoded state to that of a single attention head. Conv-1D, which operates on the encoded latent space regardless of the number of attention heads, is used as the Transformer encoder in this study in place of a single feedforward layer. A Softmax prediction is computed from the weighted sum of all layers in the multi-head attention architecture (shown in Figure 4) and is given as

(2) $$MultiHead\left(Q,K,V\right)=\left[head_{1};head_{2};\ldots head_{m}\right]W^{O}$$

(3) $$head_{i}=Attention\left({\mathrm{QW}}_{i}^{Q},{\mathrm{KW}}_{i}^{K},{\mathrm{VW}}_{i}^{V}\right)$$

where ${\mathrm{QW}}_{i}^{Q}$, ${\mathrm{KW}}_{i}^{K}$, and ${\mathrm{VW}}_{i}^{V}$, are learnable parameter matrices.

**Figure 5:**
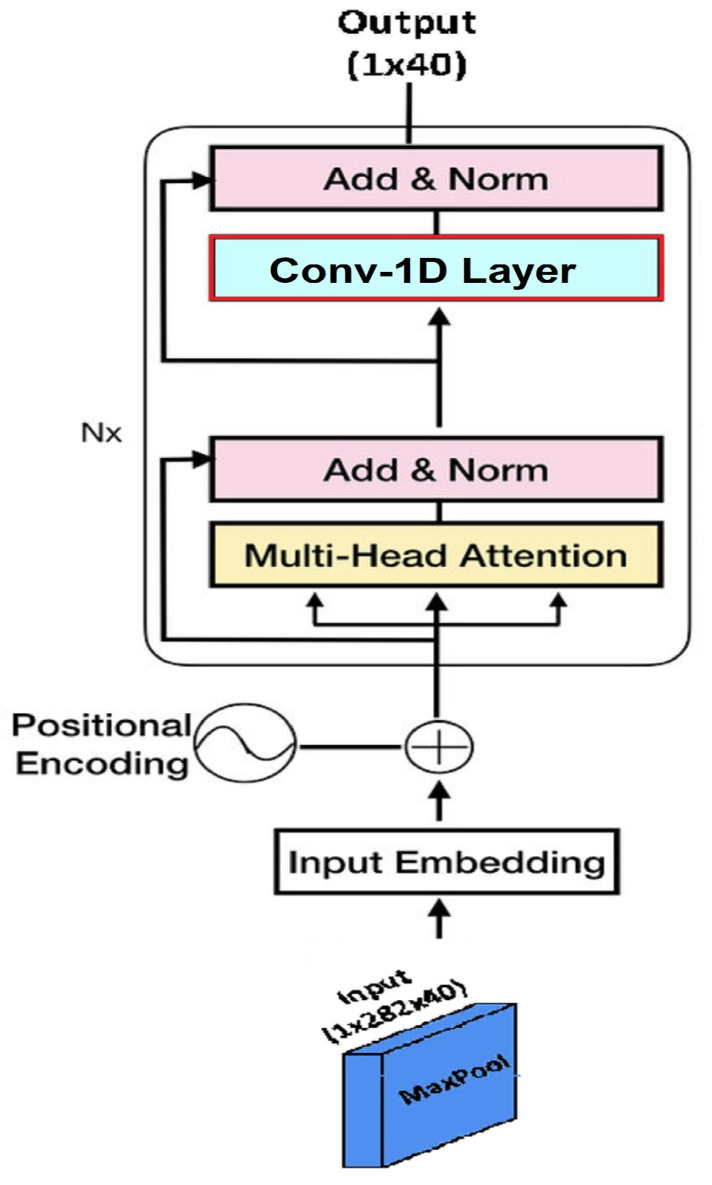
Transformer encoder architecture with input and output feature dimensions.

Four identical stacked blocks of the Transformer encoder are used to classify various emotions; each block is composed of one multi-head self-attention layer with a fully connected feedforward layer. A skip connection and a normalization layer are included subsequent to the multi-head self-attention layer. After the feedforward layer, a skip connection is created, followed by normalization. With those output by the multi-head self-attention layer, the skip connection adds the original embeddings. The normalization layer is similar to batch normalization; however, unlike batch normalization, adapted to sequential inputs, the norm layer is also applied during testing. The combined embeddings from the residual connection are subjected to the norm layer. Figure 5 depicts the design of the Transformer encoder, replacing the single feedforward layer with the Conv-1D layer.

## 5. Experimentation

This section experimentally examines the proposed CTENet model for SER and demonstrates its efficiency. We conducted extensive experiments by using the standard REVDESS dataset, an acted speech emotions dataset for SER. In addition, the IEMOCAP dataset was used to examine the performance across different databases. The performance of the proposed CTENet model has been evaluated with other state-of-the-art (SOTA) SER models that are reported in the recent literature. We also conducted an ablation learning study to confirm the multi-head attention performance in the CTENet model for SER. A complete description of the speech emotion datasets, model training/testing/validation, and emotion recognition output with discussion is given in the following sections.

### 5.1. Datasets

**Figure 6:**
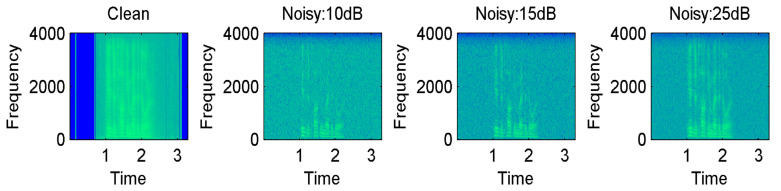
Spectrograms after adding white noise: 10 dB, 15 dB, and 25 dB.

**Figure 7:**
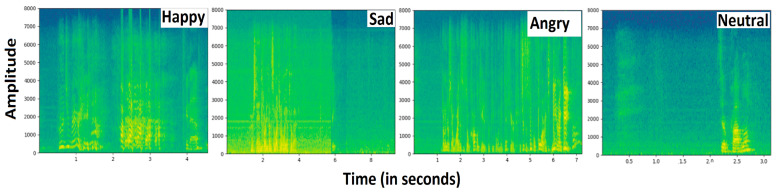
Spectrograms of various speech emotions.

The Ryerson Audiovisual Dataset of Emotional Song and Speech (RAVDESS) [83] is a new English-language scripted emotional corpus, proposed in 2018. The RAVDESS is the most popular emotional corpus and is largely used to recognize emotions in songs and speech signals. This corpus is composed of 8 emotions recorded by 24 professionals of both genders (12 females and 12 males) to produce scripts with changed emotions. Recently, the speech part of the RAVDESS corpus has been frequently utilized in comparative analysis, demonstrating the model’s generalization to many emotions. The RAVDESS speech corpus contains 1440 audio files, which are recorded at a sampling rate of 48 kHz. Since the RAVDESS speech corpus is small and is prone to overfitting, it is used exclusively with highly parameterized DNN models such as the CTENet model. Therefore, we augmented the RAVDESS speech corpus. Producing new samples is a difficult task, so we added white noise to the speech signals. The addition of white noise not only masked the effect of random noise present in the training set but also created pseudo-new training samples, which counterbalanced the impact of inherent noise in the speech corpus. Moreover, the RAVDESS corpus is extremely clean and this augmentation also evaluated the predictions of the CTENet model on noisy speech data. Note that noise addition was applied for training data only. No noise was added to the testing data on which we made emotional predictions. The spectrograms of the speech utterances from the RAVDESS corpus after adding white noise are shown in Figure 6. The details of the RAVDESS corpus are illustrated in Table 2. Interactive Emotional Dyadic Motion Capture (IEMOCAP) [84] is a speech emotions corpus provided in the English language and recorded at the University of Southern California (SAIL LAB). The corpus was recorded by 10 professional actors in five separate sessions, where each session was recorded by one male and one female actor. The corpus is composed of audio–visual files of 12 h each, where each recorded utterance has a 3.5 s length on average, comprising different emotions. This study considers five emotions, namely happiness, sadness, anger, calm, and fear, from the IEMOCAP corpus. Table 3 gives details of the speech emotions, audio file quantity, and contribution rate of each emotion. The spectrograms of various speech emotions, including happiness, sadness, anger, and neutrality, are plotted in Figure 7.

### 5.2. Model Training, Architecture, and Features

The CTENet model for SER provides outscored results in terms of emotion recognition using MFCC spectrograms. The proposed CTENet model was tested over two benchmark speech emotion datasets (RAVDESS and IEMOCAP). The speech signals were transformed into MFCC coefficients representing an utterance as a grayscale image, an appropriate 2D representation for CNN models. Adam was used to optimize the model, with a cross-entropy loss function for 200 epochs. Utterance-level extensive experiments were performed to observe the significance of the CTENet model. We followed a 80%–20%–20% training/testing/validation ratio during the experiments. Various evaluation metrics were used to examine the prediction performance of the models, such as accuracy, the F1 score, precision, and recall. We trained the CTENet models on two datasets and examined them from different aspects to demonstrate their advantages.

The CTENet model contains two parallel convolutional blocks. Each block contains a Conv-2D layer followed by batch normalization, leaky ReLU, max pooling, and dropout layers, respectively. The input and output channel dimensions in the first convolutional layer are 1 and 16, whereas the stride and kernel sizes are set to (1 × 1) and (3 × 3), respectively. The second convolutional layer is the same as the first, but with a different output dimension (32) and max-pooling kernel size (4 × 4). The third convolutional layer is similar to the second but with a different output dimension (64). A 32-dimension minibatch size and 0.20 dropout rate are used in the CTENet model. The second convolutional layer follows an identical architecture. In both parallel CNN blocks, the feature maps are batch-normalized before applying the leaky ReLU activation. The input feature map is zero-padded 1 to every convolutional layer to obtain the same tensor shape. At the end of the first convolutional layer in each parallel CNN block, the output feature map is max-pooled with a kernel of size (2 × 2) with stride 2, which takes MFCC pixels producing a (20 × 141) output feature map. The non-overlapping max-pooling kernel reduces the output dimension to the input dimension/kernel size. The output channel’s dimension is then expanded to 16, creating an output (16 × 20 × 141) feature map. In the next two convolutional layers of each CNN, the block has a max-pooling kernel size (4 × 4) with stride 4. The feature maps at the end of the second and third convolutional layers are (32 × 5 × 35) and (64 × 1 × 8), respectively. The output convolutional embedding length for both parallel CNNs is (1 × 512). Complete details are provided in Table 4.

The input MFCC coefficient maps to the Transformer encoder are max-pooled (1 × 4) with stride 4 to obtain a (1 × 40 × 70) output feature map. Therefore, the input to the Transformer embedding is (40 × 70). The final Transformer embedding length is (1 × 40). The fully connected dense layer concatenates the final embedding length from the convolutional and Transformer blocks as (512 + 512 + 40) and is used as input to the dense layer with 1064 nodes. The output from the final layer is a linear k-dimension array, which is applied to the log Softmax layer to recognize emotions. The output for RAVDESS is an 8-d array, whereas, for IEMOCAP, it is a 5-d array. The final output R is fed to the fully connected dense layer, followed by the log Softmax layer to calculate the probabilities of emotion class C, given as

(4) $$X=R+ReLU\left(RW_{R}\right)+b_{R}$$

(5) $$P=softmax\left(ZW_{Z}\right)+b_{Z}$$

while $b_{Z}\in R^{C}$, $W_{R}\in R^{d_{2}\times d_{2}}$, $W_{Z}\in R^{d_{2}\times C}$, and $b_{R}\in R^{d_{2}}$ are trainable parameters, whereas $X\in R^{d_{2}\times N}$, and $X\in R^{C\times N}$. The most likely predicted emotion class can be selected as

(6) $${\hat{z}}^{\left(k\right)}=argmax\left(P^{\left(k\right)}\right)$$

where $\left(P^{\left(k\right)}\right)\in R^{C}$ and ${\hat{z}}^{\left(k\right)}\in R^{1}$ are the probabilities of each emotion class. In the training, the cross-entropy loss function is used, given as

(7) $$Loss=-\sum_{i}^{M}y_{i,C}^{k}log_{2}\left(y_{i,C}^{k}\right)$$

while *M* indicates the number of classes (happy, angry, sad, etc.)

### 5.3. Baseline Models

For the comparison, we selected the following SOTA baseline models to extensively evaluate the performance of the CTENet model. Att-Net [68] is a robust SOTA lightweight self-attention model for SER, where a dilated CNN uses channel and spatial attention for the extraction of cues from the input tensors. The SVM ensemble model with a Gaussian kernel [50] is a standard benchmark used for SER comparison. The 1D-CNN [74] architecture is used, which extracts MFCC features and uses the trained 1D-CNN for emotion identification. The context-aware representations are used for emotion recognition. DeepNet [60] learns deep features and employs a plain rectangular filter with a new pooling scheme to achieve more effective emotion discrimination. The other SOTA models include GResNets [85]; SER using 1D-Dilated CNN, which is based on the multi-learning trick (MLT) [86]; and the CNN-BLSTM-based SER method from [57].

## 6. Results and Discussion

In this section, the results of the CTENet model in terms of various measures are first presented. Then, we compare the CTENet model with other SOTA models for SER using the RAVDESS and IEMOCAP corpora.

**Figure 8:**
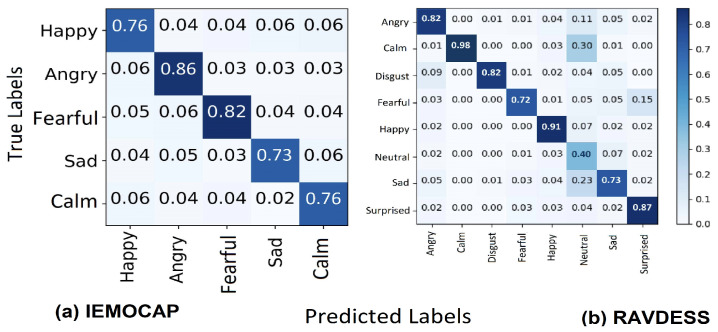
Confusion metrics. (**a**) IEMOCAP dataset, (**b**) RAVDESS dataset.

We examined the emotion recognition performance of the suggested CTENet model and utilized various measures to evaluate the model, such as recognition accuracy, precision, F1 scores, and recall. The confusion matrix plots of the model visualized the model performance in terms of actual and predicted labels for each emotion class. In addition, we conducted an ablation study for different emotions and achieved results with different models. The results of CTENet for the RAVDESS and IEMOCAP datasets are illustrated in Table 5 with regard to recognition rates for each emotion class. We present the recognition accuracy of CTENet for each speech emotion from the RAVDESS and IEMOCAP datasets (W.Acc indicates weighted accuracy, whereas UW. Acc denotes unweighted accuracy). In addition, the confusion matrices visualize the testing sets in Figure 8. For RAVDESS (8-way), the simulation results in Table 5 show that CTENet obtains improved recognition accuracy in individual speech emotion recognition tasks at most times, exclusively for the happy, calm, surprised, and angry emotions. Meanwhile, we find that CTENet confuses the calm and angry emotions with the neutral and disgust emotions in a few cases (as demonstrated in Figure 8a). Consequently, the CTENet model requires us to learn more about anger and disgust. The lowest recognition accuracy is obtained for the neutral emotion, since the neutral emotion is under-represented in the RAVDESS dataset (6.67% of the dataset). For the IEMOCAP (5-way) dataset, improvements in recognition performance can be seen for most emotion classes, as shown in Table 5. Specifically, anger and fear outscore other speech emotions, including happiness, sadness, and calm. This can be attributed to the better ability of the CTENet model to classify features that are important for emotional discrimination. Meanwhile, we find that a few emotions are confused with others in some cases (as shown in Figure 8b).

**Figure 9:**
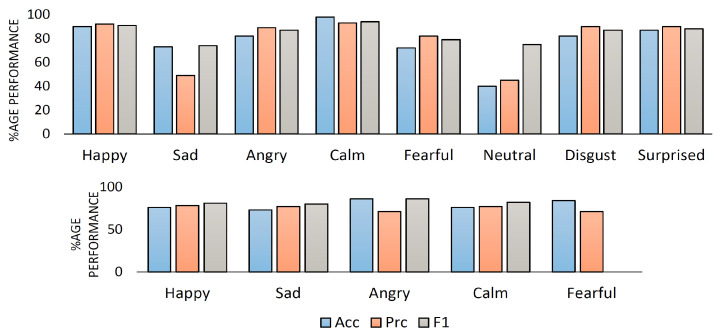
CTENet percentage performance: accuracy (Acc), precision (Prc), and F1 score using RAVDESS and IEMOCAP datasets.

Table 6 and Table 7 describe the experimental results of CTENet model prediction in terms of overall model precision and the F1-score for the RAVDESS and IEMOCAP datasets. The experimental results show that CTENet obtains improved F1 accuracy and precision in the individual speech emotion recognition tasks for most instances, exclusively for the happy and calm emotions, for both the RAVDESS (8-way) and IEMOCAP (5-way) datasets. We confirmed the robustness of CTENet over the two standard datasets, and it achieved 78.75% precision for RAVDESS and 74.80% precision for IEMOCAP. Furthermore, CTENet achieved 84.38% F1 for RAVDESS and 82.20% F1 for IEMOCAP, respectively. The CTENet accuracy for the two datasets was 82.31% and 79.42%, respectively. Figure 9 visualizes the complete performance of CTENet for both datasets in terms of precision, accuracy, and F1 scores, respectively.

To show the importance of the multi-head attention Transformer (MHAT) encoder in CTENet, we present Table 8, which demonstrates the accuracy, precision, and F1 scores for speech emotions achieved with CTENet without MHAT and with the MHAT encoder, respectively. The experimental outcomes indicate the significance of MHAT inclusion in CTENet, where the recognition results are enhanced considerably. On average, the accuracy, precision, and F1 scores are improved by 7.29%, 5.13%, and 3.26%, respectively, with MHAT. The accuracy is improved from 70.32% with the RAVDESS dataset to 78.0%, and from 70.32% to 79.0% with the IEMOCAP dataset. In addition, the F1 score is improved from 80.40% with the RAVDESS dataset to 84.37%, whereas it changes from 79.65% to 82.20% with the IEMOCAP dataset.

The proposed CTENet model demonstrated improved generalization during the experiments and evaluations for both datasets, and it obtained better emotion recognition accuracy with a low computational cost. In brief, we can assume that the proposed CTENet model for SER is accurate and computationally less complex. Consequently, it is able to examine human behaviors and emotions. Moreover, with the lightweight framework, this model is appropriate for real-time applications since it requires less training time. Table 9 gives the training time and model size (in Mb). We compared the training time and model size with those of other SER frameworks, including DS-CNN [51], CB-SER [57], and AttNet [68], for comparison. The experiments proved that the CTENet model is lightweight (compact model size of 4.54 Mb), generalizable, and computationally less expensive, and it requires less processing time to recognize emotions, which indicates the appropriateness of the model for real-world applications. The processing time is significantly minimized as the simultaneously expanded filter depth and feature map reduction provide an expressive hierarchical feature representation at the minimum computational cost. The total trainable parameters are 222,248 for the CTENet model.

### Comparison with Existing Models

**Figure 10:**
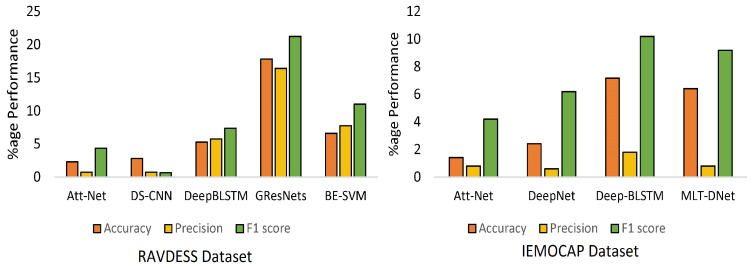
CTENet performance over SOTA for RAVDESS and IEMOCAP datasets.

To confirm the effectiveness of the presented method, we compared CTENet with SOTA baseline benchmarks on the RAVDESS (8-way) and IEMOCAP (5-way) datasets. The SOTA baseline benchmarks included Att-Net, ensemble SVMs, 1D-CNN, BC-LSTM, ConvLSTM, and DeepNet. This section first compares CTENet with the SOTA baseline benchmarks in terms of the overall performance using accuracy, precision, and F1. After this, we compare the recognition accuracy, precision, and F1 for individual emotions. Table 10 shows a comparison of CTENet with the SOTA baseline benchmarks on the RAVDESS and IEMOCAP datasets. The experimental results show the effectiveness of CTENet. For the RAVDESS dataset, CTENet achieves 82.31% accuracy, which indicates an improvement of 2.31% over Att-Net, 2.81% over DS-CNN, and improvements other SOTA models given in Table 10. In addition, for the IEMOCAP dataset, the CTENet achieves 79.42% accuracy, indicating an absolute improvement of 6.92% over Deep-BLSTM, 2.42% over DeepNet, and improvements over other SOTA models with reasonable margins. CTENet surpasses BE-SVM, GResNets, and Deep-BLSTM and improves the precision by 7.75%, 16.43%, and 5.75% on an absolute scale for the RAVDESS dataset. For the IEMOCAP dataset, CTENet outperforms the SOTA models, except for DS-CNN, which improves the precision by 12%. In terms of the F1 score, CTENet consistently achieves the highest percentage improvements. The overall F1 for CTENet is 82.20%, which is 6.0%, 10.0%, and 5.0% higher than that of DeepNet, Deep-BLSTM, and MLT-DNet for the IEMOCAP dataset. On the other hand, for the RAVDESS dataset, the CTENet achieves an 84.37% F1 score, which is 7.37% higher than that of Deep-BLSTM and 21.26% higher than GResNets. Figure 10 shows the detailed performance of CTENet over the SOTA models [87].

## 7. Conclusions and Recommendations

In this paper, we describe the combination of spatial and temporal feature representations of speech emotions by parallelizing CNNs and a Transformer encoder for SER. We extract the spatial and temporal features with parallel CNNs and the Transformer encoder from the MFCC spectrum. In the CTENet model, MFCCs are used as grayscale images, where the width is the time scale and height is the frequency scale. The experimental results on two popular benchmark datasets, RAVDESS and IEMOCAP, validate the usefulness of the CTENet model for SER. Our model achieves better experimental results over state-of-the-art models for speech emotion recognition, with overall accuracy of 82.31% and 79.80% for the benchmark datasets. Furthermore, the experimental results for different speech emotion classes show the effectiveness of the spatial and temporal feature fusion. The experimental results show the importance of MHAT inclusion in CTENet, where the emotion recognition results are improved significantly. The experimental results also prove that CTENet is compact (4.54 Mb) and computationally less costly, and requires less processing time to recognize different emotions, indicating the appropriateness of CTENet for real-world applications. With few entries in the datasets, the model sometimes overfits; however, we can fine-tune the model to avoid overfitting, such as by applying dropout regularization. It is also recommended to increase the database entries for better results and optimized model parameters.

The present study provides acceptable accuracy; however, a further improvement in accuracy can be achieved if the model architecture is further refined, e.g., a more effective feature extractor can be adopted. Different feature sets can be combined for more robust training features. Further, besides temporal and spatial features, we aim to add modalities to further increase the recognition accuracy using modality cues. In addition, we will apply recently introduced models to achieve state-of-the-art SER results.

## Figures and Tables

**Table 1:** Summary of SER-related literature.

Ref #	DNN Model	Model Input	Input Features	Accuracy
[74]	CNNAtt-Net	Spectrograms	Spatial Features	80.00%
[71]	SVM-DBN	MFCC	Prosodic + Spectral	80.11%
[63]	CNN-BLSTM	Spectrograms	Spatial + Temporal	77.02%
[56]	Bagged SVM	Spectrograms	Spectral Features	75.79%
[66]	Lightweight CNN	Spectrograms	Spectral Features	75.01%
[69]	ConvLSTM	Spectrograms	Spectral Features	75.00%
[60]	1D-CNN-FCN	MFCC	Prosodic + Spectral	72.19%
[82]	1D-CNN	MFCC + Chromagram + Spectrogram	Spatial Features	71.61%
[78]	TDNN-LSTM-Attn	Raw Spectra	Spectral Features	70.11%
[72]	DS-CNN	Raw Spectra	Spatial Features	70.00%
[55]	Voting LMT	Spectrograms	Spectral Features	67.14%
[73]	RNN-Attn	Raw Spectra	Acoustic + Temporal	63.50%
[70]	BLSTM-CNN	MFCC	Prosodic + Spectral	57.84%

**Table 2:** Details of the emotions, audio files, and percentage contributions of the RAVDESS database for the CTENet model.

Emotion	Audio Files	Contribution
Happiness	192	13.33%
Sadness	192	13.33%
Anger	192	13.33%
Calm	192	13.33%
Fear	192	13.33%
Neutral	96	6.667%
Disgust	192	13.33%
Surprise	192	13.33%

**Table 3:** Details of the emotions, audio files, and percentage contributions of the IEMOCAP database for the CTENet model.

Emotion	Audio Files	Contribution
Happiness	1636	24.33%
Sadness	1084	16.12%
Anger	1103	16.40%
Calm	1700	25.28%
Fear	1200	17.84%

**Table 4:** CNN model architecture with input/output dimensions, filter size, and stride.

Layer	Input Dim	Padding	Output Dim	Filter Size	Output Dim	Maxpool, Stride	Output Dim
1	(1 × 282 × 40)	1	(1 × 284 × 42)	(1 × 3 × 3)	(16 × 40 × 282)	(2 × 2), 2	(16 × 20 × 141)
2	(16 × 20 × 141)	1	(16 × 22 × 143)	(16 × 3 × 3)	(32 × 20 × 141)	(4 × 4), 4	(32 × 5 × 35)
3	(32 × 5 × 35)	1	(32 × 7 × 37)	(32 × 3 × 3)	(64 × 5 × 35)	(4 × 4), 4	(64 × 1 × 8)
Flatten (64 × 1 × 8); final convolutional embedding length (1 × 512).							

**Table 5:** Speech emotion recognition in terms of accuracy (in %) for the RAVDESS and IEMOCAP corpora.

Datasets	Speech Emotions									
	**Happy**	**Sad**	**Angry**	**Calm**	**Fearful**	**Neutral**	**Disgust**	**Surprised**	**W.Acc**	**UW.Acc**
RAVDESS	90.1	73.2	82.2	98.1	72.3	40.2	82.1	87.1	79.3	78.2
IEMOCAP	76.6	73.8	86.6	76.1	84.2	-	-	-	80.3	79.5

**Table 6:** CTENet model prediction performance (in %) using RAVDESS dataset.

Happy		Sad		Angry		Calm		Fearful		Neutral		Disgust		Surprised	
**Prc**	**F1**	**Prc**	**F1**	**Prc**	**F1**	**Prc**	**F1**	**Prc**	**F1**	**Prc**	**F1**	**Prc**	**F1**	**Prc**	**F1**
92	91	49	74	89	87	93	94	82	79	45	75	90	87	90	88
Model Accuracy: 82.31%															

**Table 7:** CTENet model prediction performance (in %) using IEMOCAP dataset.

Happy		Sad		Angry		Calm		Fearful	
**Prc**	**F1**	**Prc**	**F1**	**Prc**	**F1**	**Prc**	**F1**	**Prc**	**F1**
78	81	77	80	71	86	77	82	71	82
Model Accuracy: 79.42%									

**Table 8:** CTENet prediction performance (in %) with and without multi-head attention Transformer.

Model Input	Database	Neural Architecture	Accuracy	Precision	F1 Score
MFCC Spectrum	RAVDESS	CTENet without MHAT	72.10	73.34	80.40
	IEMOCAP		70.32	69.95	79.65
MFCC Spectrum	RAVDESS	CTENet with MHAT	78.00	78.75	84.37
	IEMOCAP		79.00	74.80	82.20

**Table 9:** CTENet model’s computational size and processing time.

Models	RAVDESS	IEMOCAP	Model Size
DSCNN [51]	2400 s	2640 s	34.5 MB
CB-SER [57]	6250 s	10,452 s	125 MB
AttNet [68]	1900 s	2100 s	14.4 MB
CTENet	1600 s	1900 s	4.54 MB

**Table 10:** Comparison of CTENet with benchmarks.

			RAVDESS Dataset			IEMOCAP Dataset		
**Ref#**	**Benchmarks**	**Input Features**	**Accuracy**	**Precision**	**F1 Score**	**Accuracy**	**Precision**	**F1 Score**
[56]	BE-SVM	Spectral Features	75.69	74.00	73.34	-	-	-
[85]	GResNets	Spectral Features	64.48	65.32	63.11	-	-	-
[86]	MLT-DNet	Spatial Features	-	-	-	73.01	74.00	73.00
[57]	Deep-BLSTM	Spatial + Temporal	77.02	76.00	77.00	72.50	73.00	72.00
[74]	1D-CNN	Spectral Features	71.61	-	-	64.30	-	-
[66]	DS-CNN	Spatial Features	79.50	81.00	84.00	78.75	86.00	82.00
[60]	DeepNet	Spatial + Temporal	-	-	-	77.00	76.00	76.00
[68]	Att-Net	Spatial Features	80.00	81.00	80.00	78.00	78.00	78.00
Our	CTENet	Spatial + Temporal	82.31	81.75	84.37	79.42	74.80	82.20

## Data Availability

The datasets are available at IEMOCAP: https://sail.usc.edu/iemocap/, accessed on date 21 January 2023, RAVDESS: https://www.kaggle.com/datasets/uwrfkaggler/ravdess-emotional-speech-audio, accessed on date 21 January 2023.
